# Outcome of surgical resection of localized gastrointestinal stromal tumors: our experience

**DOI:** 10.1186/1471-2482-13-S1-A10

**Published:** 2013-09-16

**Authors:** Michele Danzi, Luciano Grimaldi, Massimiliano Fabozzi, Umberto Robustelli, Roberta Danzi, Bruno Amato, Stefano Reggio

**Affiliations:** 1 Department of Specialized Surgery, Division of Gastrointestinal Surgery Rehabilitation of Election and Emergency. “Federico II” University, Naples, Italy; 2 Department of General Surgery . “U. Parini” Hospital, Aosta, Italy; 3 Emergency and General Surgery “AORN Cardarelli” Naples, Italy; 4 Department of Diagnostic Imaging and Radiotherapy. “Federico II” University, Naples, Italy

## Background

Gastrointestinal stromal tumors (GISTs) constitute the most common non epithelial neoplasm that occur within the gastrointestinal tract with a world wide annual incidence of 8-14/million. They are usually located in the upper gastrointestinal tract particularly in the stomach (60%), in the small bowel (30%), esophagus (5%) and rectum (5%). Gist(s) origin from sporadic mutations within the tyrosine-kinase receptors of the interstitial Cajal cells. The clinical presentation of Gist(s) is quite heterogeneous [1]. The most common associated symptoms are abdominal pain, bleeding, gastro-enteric outlet obstruction. Modern cross-sectional imaging studies include TC and/or magnetic resonance imaging in combination with upper endoscopy. Upper endoscopy with ultrasonography (EUS) is an useful tool to pick tissue for diagnosis for cytology (FNA) or tru-cut biopsy. In addition EUS can show the depth of penetration through the layers of the gastro-enteric wall and origin of these neoplasm.

Routinary biopsies increase the risk of tumor spillage or hemorrhage with consequent higher rate of recurrence or disseminated sarcomatosis therefore only selective biopsies are recommended [2].

Complete surgical resection is the only curative therapy of Gist without metastasis. Surgery of large Gists may result technically difficult. However debulking before molecular therapy or reducing the size with neoadjuvant therapy by imatinib can give longer survival and better results [3].

In this study we report our experience on surgical treatment of 16 patients with localized gastrointestinal stromal tumors.

## Methods

From 2003 to 2011, 16 cases of gastrointestinal stromal tumors underwent to surgery in the Department of Gastrointestinal Surgery of Naples University “Federico II”. There were 7 males and 9 females with a mean age of 63.2 years (range 53-84 years). All the patients underwent surgery for localyzed Gist.

Diagnosis was based on histo-pathological appearance and was confirmed by positive immunohistochemical staining for c-kit protein CD 117, CD34, SMA, S100 were also performed.

All patients underwent surgical resection. On the basis of size of the primary tumor, mitotic index an Fletcher’s criteria, the tumors were classified as very low, low, intermediate and high risk 4.

Survival and relapse rate were considered. For survival analysis was used the Log-rank test. Statistical significance of frequencies was performed with χ square test and T test for means comparison.

## Results

From June 2003 to November 2011, 16 patients were diagnosed with Gist 6 patients were referred after diagnosis defined elsewhere. 8 patients were symptomatic and 4 of them presented with abdominal mass, 1 had as first symptom gastrointestinal bleeding, 1 presented with gut occlusion, 2 had appetite and weigth loss.

The organs affected were primary the stomach in 9 cases and small bowel in 7. The NIH class of risk at the pathological evaluation after surgery was high 4/16 (25%), intermediate 2/16 (12.5%), low 7/16 (44%), very low 3/16 (9%). On histopathology 98% of cases were positive for CD 117 immunohistochemistry, CD 34 was 81%, SMA 45%, S100 28%.

Of 16 who underwent surgical treatment, 13 (81%) underwent complete resection (R0), 2 had R1 resection, in these latter patients, tumor was located in small bowel. Four of the patients who had a complete resection later developed local recurrence. Thyrosine kinase inhibitor therapy was given to patients with residual disease R1 resection, in case of relapse after primary surgical resection. Of 6 patients who received tyrosine kinase inhibitor therapy (400 mg/day), 4 had no progression disease 1 had progressive disease and 1 had no complete response.

We analyzed disease free survival at five years and relapse rate in all patients. Twelve patients belonging to low and intermediate risk group had disease free survival of 90%.

In high risk group patients, disease free survival was 50% at five years. In four patient in the high risk group, we observe 2 relapses of disease with massive progression of disease due to liver metastatic out peritoneal metastasis. These patients died 26 months and 34 months after surgical operation. Low-risk patients did not receive any treatment and were all disease free at the follow-up. One of two intermediate- risk patients had recurrence in the peritoneum 32 months after surgery and imatinib was increased to 600 mg/day.

## Conclusions

This study consisted of heterogeneous groups of patients. The mean age of the patients was 63.2. The most common site of Gist was the stomach in 9 cases and the small bowel in 7 cases. The mainstay treatment of Gist is surgical excision. The global five year disease free survival was 81%. When complete resection is not possible, target therapy with tyrosine-kinase is the choice treatment. In our study only few patients belonged to the high risk group (4/16). We found that the failure cases occurred in the original high and intermediate risk category. This result may suggest an essential role of postoperative adjuvant treatment in the high risk patients. De Matteo 5 suggested adjuvant imatinib treatment may improve disease free survival after surgical resection of primary gastrointestinal stromal tumor. In terms of survival a good outcome is expected when a complete tumor excision is obtained. Moreover patient who were in the low risk group, had more possibility to have a complete tumor removal and survival probability is more than 90%. In conclusion the advent of new molecular markers has led to improve local control and improve disease-free survival. Prognosis is mainly influenced by tumor respectability risk-categorization and response to tyrosine kinase treatment. However, large, randomized prospective trials are still needed to address the best therapeutic modalities and improve survival.

## References

[B1] Demetri GD Von Mehren M Task Force report:update on the management of patients with gastrointestinal stromal tumors J Natl Compr Canc Netw 2010 8 suppl.2 21 41 10.6004/jnccn.2010.0116 PMC4103754 20457867

[B2] Martella B Cardin F Lorenzetti R Terranova C Amato B Militello C Local recurrence of gastric cancer after total gastrectomy: An unusual presentation BMC Surgery 2012 12 SUPPL. 1 S28 23173846 10.1186/1471-2482-12-S1-S28 PMC3499257

[B3] Gold JS De Matteo RP Combined surgical and molecular therapy: the gastrointestinal stromal tumor model Ann Surg 2006 244 176 184 10.1097/01.sla.0000218080.94145.cf 16858179 PMC1602162

[B4] Gold JS van der Zwan SM Gönen M Maki RG Singer S Brennan MF Antonescu CR De Matteo RP Outcome of metastatic GIST in the era before tyrosine kinase inhibitors Ann Surg Oncol 2007 14 1 134 142 17080234 10.1245/s10434-006-9177-7

[B5] Angelini S Ravegnini G Fletcher JA Maffei F Hrelia P Clinical relevance of pharmacogenetics in gastrointestinal stromal tumor treatment in the era of personalized therapy Ann Surg Oncol 2007 14 8 941 956 10.2217/pgs.13.63 23746188

